# Research advances in the adjunctive diagnosis of acute myeloid leukemia

**DOI:** 10.3389/fonc.2025.1634935

**Published:** 2025-10-07

**Authors:** Wentao Xie, Xinye Jiang, Jingying Huang, Mingwei Qin, Zhisheng Bi

**Affiliations:** ^1^ School of Biomedical Engineering, Guangzhou Medical University, Guangzhou, China; ^2^ Department of Emergency, the Second Affiliated Hospital, Guangzhou Medical University, Guangzhou, China

**Keywords:** acute myeloid leukemia, blood smear image, flow cytometry, genetic analysis, artificial intelligence

## Abstract

Acute myeloid leukemia (AML) is a highly heterogeneous malignant hematological neoplasm. Although standard diagnostic procedures have been established, traditional methods still face limitations with regard to efficiency, accuracy, and standardization. In recent years, artificial intelligence (AI) has demonstrated notable advantages in medical image analysis, flow cytometry interpretation, and genetic data modeling, offering new approaches for adjunctive diagnosis of AML. This review systematically summarizes recent research advances in adjunctive diagnosis of AML, categorizing current AI-based approaches based on data modality into three groups: blood smear image analysis, flow cytometry data interpretation, and genetic data modeling. We focus on the application strategies, diagnostic performance, and limitations of these approaches. Studies have shown that AI not only enhances diagnostic efficiency and reduces subjective bias, but also holds promise in identifying novel biomarkers. Nevertheless, current models still suffer from limited generalizability and insufficient clinical interpretability. Future efforts should prioritize data standardization, improve model transparency, and facilitate the seamless integration of AI systems into clinical workflows to support precision diagnosis and treatment of AML.

## 1 Introduction

**Figure 1:**
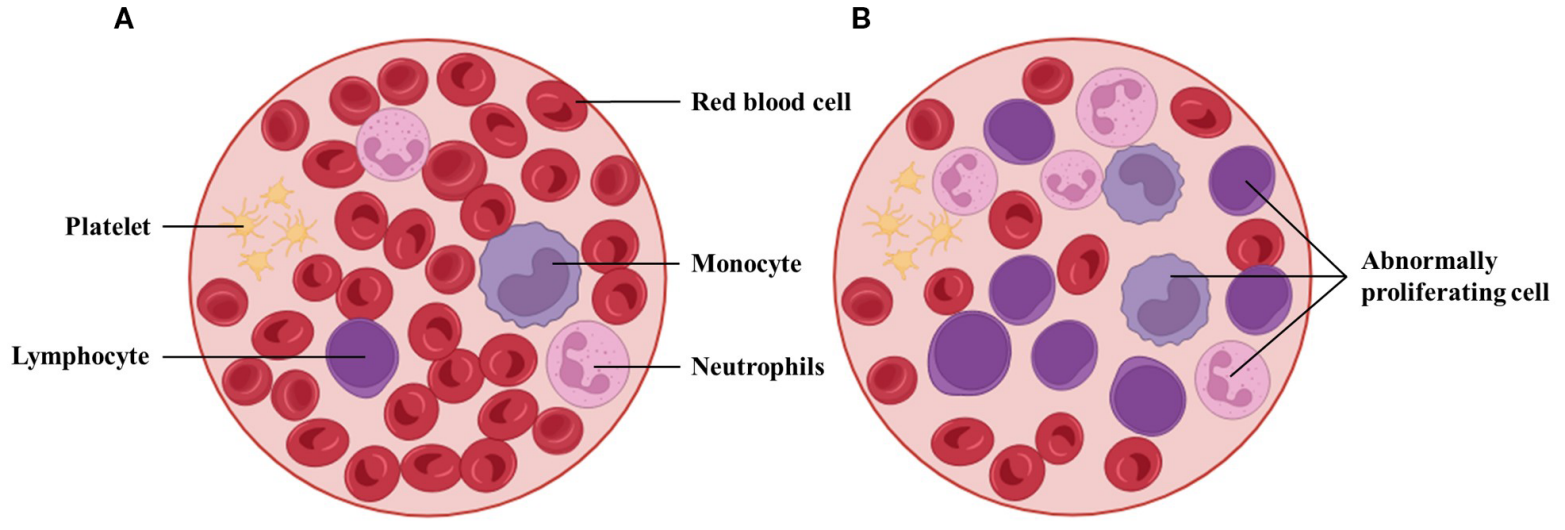
Morphological comparison between peripheral blood from a healthy individual and a patient with AML. **(A)** Normal smear with abundant mature erythrocytes and leukocyte subtypes. **(B)** AML smear with reduced mature cells and increased blasts. Such differences underpin AI-assisted blast detection and triage in hematology workflows, though performance depends on stain/scan standardization and external validation.

Acute myeloid leukemia (AML) is a hematopoietic malignancy characterized by marked molecular and clinical heterogeneity, accounting for approximately 80% of adult acute leukemia cases (1). According to data from the Global Burden of Disease Project, the global burden of AML has increased substantially between 1990 and 2021, with the annual incidence rising from 79,372 to 144,645 cases, and annual mortality increasing from 74,917 to 130,189 deaths (2). Pathologically, AML is driven by the accumulation of genetic alterations in myeloid progenitor cells, resulting in impaired differentiation and uncontrolled proliferation (see **Figure 1**), ultimately leading to hematopoietic failure (3). Clinically, AML often presents with nonspecific symptoms such as anemia, fever, and fatigue (4), yet progresses rapidly and is difficult to manage (5, 6). Notably, even after initial treatment, residual leukemic cells known as minimal residual disease (MRD) may persist, representing a key factor contributing to disease relapse (7). Overall, AML is associated with poor prognosis, with a 5-year survival rate of approximately 30%, and less than 10% in patients over the age of 65 (8). These challenges highlight the urgent need for more accurate diagnostic modalities, robust risk stratification frameworks, and individualized treatment strategies to improve clinical outcomes.

In recent years, artificial intelligence (AI) technologies, including machine learning and deep learning, have shown remarkable potential in recognizing complex patterns, analyzing high-dimensional data, and facilitating clinical decision-making (9, 10). Within the field of medical image analysis, AI algorithms have achieved significant success in tasks such as lesion detection, organ segmentation, and diagnostic assistance. In some scenarios, their performance has matched or even surpassed that of experienced physicians (11, 12). Beyond imaging, AI has also been extensively applied to the analysis of multidimensional datasets, such as flow cytometry and genomic profiles, to support disease prediction, classification, prognosis assessment, and evaluation of therapeutic responses (13, 14). The integration of AI into the automated analysis of images and flow cytometry data can greatly reduce diagnostic turnaround time, which is particularly critical for conditions requiring prompt intervention, such as acute promyelocytic leukemia (APL) (15), Additionally, AI systems help reduce subjectivity in morphological and flow cytometric interpretation, thereby improving reproducibility and standardization. More importantly, AI models are capable of identifying subtle features and potential novel biomarkers that may be imperceptible to human experts, contributing to a more profound understanding of disease pathogenesis.

In this review, “adjunctive” AI-assisted AML diagnosis refers to systems that do not render an autonomous final diagnosis. Instead, they function as decision-support tools that generate data-driven analyses—such as risk scores, classification recommendations, anomaly alerts, or triage prioritization—to enhance expert judgment, improve efficiency and consistency, and integrate with existing workflows. The final diagnostic decision remains with the treating physician.

Therefore, this review systematically summarizes recent advances in adjunctive diagnosis of AML, categorized by data modality into three major areas: blood smear image analysis, flow cytometry data interpretation, and genetic data modeling. For each modality, we examine the applied strategies, diagnostic performance, and inherent limitations. Finally, we discuss current challenges and outline future directions for the integration of AI-based adjunctive diagnostic techniques into routine clinical practice.

## 2 Review methods

This review was designed as a narrative survey of research on AI applied to adjunctive diagnosis of AML. Literature searches were conducted in PubMed and Web of Science, covering publications from January 2015 to March 2025, in order to capture both early applications and the most recent advances. The following keywords and their combinations were applied: “acute myeloid leukemia,” “artificial intelligence,” “machine learning,” “deep learning,” “blood smear,” “flow cytometry,” and “genomics.” Searches were limited to studies published in English.

Inclusion criteria were (1): original research applying AI or machine learning methods to AML diagnosis, subtype classification, MRD detection, or molecular feature prediction. (2) studies based on blood smear morphology, flow cytometry data, or genetic datasets. (3) reports providing quantitative outcomes such as accuracy, sensitivity, specificity, or AUC.

Exclusion criteria were: (1) studies focused solely on therapeutic prediction, drug screening, or treatment response without diagnostic relevance; and (2) narrative reviews, editorials, or conference abstracts lacking sufficient methodological detail.

Preprints (bioRxiv/medRxiv) were included when they presented AML-specific AI diagnostic research not yet available in peer-reviewed journals; these are clearly labeled as preprints in the References. Two authors independently screened titles/abstracts, reviewed full texts for eligibility, and extracted study design, data modality, sample size/splits, AI approach, and diagnostic performance metrics.

## 3 AML diagnosis: standards and clinical practice

### 3.1 Classification criteria

The clinical classification and diagnosis of AML are primarily based on three major systems. The first is the French-American-British (FAB) classification proposed in 1976 (16), which uses a threshold of >30% blast cells in the bone marrow for diagnosis. Based on cytomorphology and cytochemical staining, AML is subdivided into eight types (M0–M7).

The second is the fifth edition of the World Health Organization (WHO) classification of hematologic malignancies (17), which lowers the diagnostic threshold to >20% blasts in the bone marrow or peripheral blood (see **Table 1**). It incorporates morphological, immunophenotypic, cytogenetic, and molecular genetic features into a comprehensive MICM (Morphology, Immunophenotype, Cytogenetics, Molecular abnormalities) framework. Notably, patients with specific genetic abnormalities such as PML::RARA or RUNX1::RUNX1T1 can be diagnosed with AML even if the blast percentage is below 20%.

**Table 1:** WHO classification of AML and required blast cell proportion for diagnosis.

WHO 5^th^ edition classification of AML	Blast percentage
AML with RUNX1::RUNX1T1 fusion AML with CBFB::MYH11 fusion AML with DEK::NUP214 fusion AML with RBM15::MRTFA fusion AML with KMT2A rearrangement AML with MECOM rearrangement AML with NUP98 rearrangement AML with mutated NPM1 AML with other defined genetic alterations	No blast threshold
AML with BCR::ABL1 fusion AML with biallelic CEBPA mutations AML, myelodysplasia-related AML with minimal differentiation AML without maturation AML with maturation Acute basophilic leukemia Acute myelomonocytic leukemia Acute monocytic leukemia Acute erythroid leukemia Acute megakaryoblastic leukemia	≥20%

The third is the International Consensus Classification (ICC) released in 2022 (18), which largely aligns with the WHO system but introduces refinements (see **Table 2**). While retaining the 20% blast threshold as a general criterion, ICC allows for AML diagnosis at ≥10% blasts in certain clinical contexts, such as therapy-related or secondary AML, or in the presence of high-risk genetic mutations. Additionally, ICC delineates precursor states such as myelodysplasia-related AML, refines mutational criteria, and introduces several high-risk biomarkers to enhance diagnostic granularity.

**Table 2:** ICC classification of AML and required blast cell proportion for diagnosis.

ICC 2022 edition classification of AML	Blast percentage
AML with RUNX1::RUNX1T1 fusion AML with CBFB::MYH11 fusion AML with DEK::NUP214 fusion AML with KMT2A rearrangement AML with MECOM rearrangement AML with BCR::ABL1 fusion AML with mutated NPM1 AML with in-frame bZIP domain CEBPA mutation	≥10%
AML with mutated TP53 AML with myelodysplasia-related gene mutations AML with myelodysplasia-related cytogenetic abnormalities AML not otherwise specified	≥20%

In addition, the guidelines issued by the European LeukemiaNet (ELN) stratify patients into favorable, intermediate, and adverse risk groups (19). They also emphasize the importance of dynamically monitoring MRD using methods such as multiparameter flow cytometry (MFC) and quantitative PCR to support early prognostic evaluation and guide individualized treatment.

The FAB/WHO/ICC/ELN taxonomies define clinically accepted ground truth labels for supervised AI studies by specifying AML diagnostic categories and ELN risk strata. These frameworks also anchor clinically meaningful endpoints—such as overall/event-free survival, MRD status, and relapse—thereby aligning model outputs with prognostic relevance. Using these standardized labels and endpoints ensures cross-study comparability and enhances the translational validity of AI results.

### 3.2 Traditional diagnostic processes

Traditional diagnosis of AML typically involves the integrated application of multiple diagnostic modalities. Initial assessments include peripheral blood tests, such as complete blood count and morphological analysis of blood smears, to detect abnormalities in cell counts and morphology (20). This is followed by bone marrow aspiration and biopsy to evaluate blast cell percentage and cytomorphological features (21). Flow cytometry is then employed for immunophenotyping, enabling the detection of surface and cytoplasmic antigen expression patterns to assist in AML subtyping and MRD monitoring (22). For specific AML subtypes, additional cytogenetic and molecular genetic testing such as chromosomal aberrations and mutations in genes like FLT3 and NPM1 is often required for refined classification and risk stratification (23).

Despite the increased diagnostic accuracy achieved through multiple tests, several challenges remain in key steps. Morphological evaluation of peripheral blood smears (PBS) and bone marrow smears (BMS) depends heavily on experienced physicians for manual interpretation, which is labor-intensive, time-consuming, and prone to subjectivity (24). The diagnostic error rate in morphological assessments can be as high as 40% (25). Flow cytometry results may vary due to differences in detection protocols, antibody panel configurations, and analytical standards across laboratories, affecting reproducibility. Molecular testing, meanwhile, often requires expensive equipment and specialized reagents, with long turnaround times and high demands on data interpretation (26). Moreover, diagnostic workflows differ across clinical centers, and for AML patients, even a 24-hour delay in initiating treatment can significantly impact prognosis (27).

## 4 Adjunctive diagnostic of AML based on blood smear image data

### 4.1 Morphological analysis

**Figure 2:**
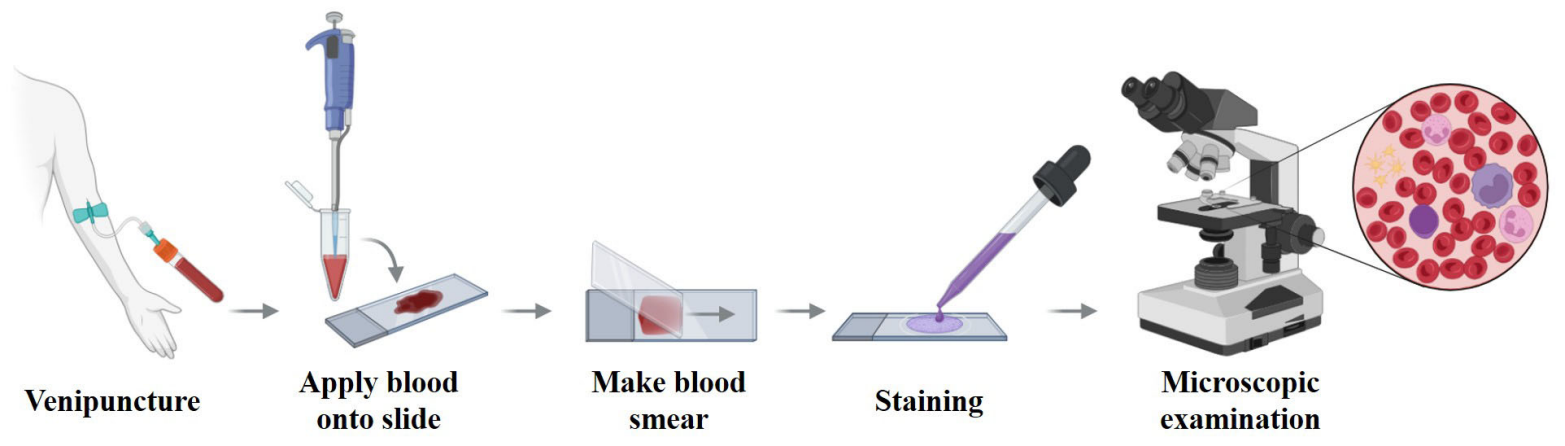
Workflow of peripheral blood smear preparation and microscopic examination. Venous blood is applied to a glass slide, spread to form a thin monolayer, air-dried, and stained (e.g., Wright–Giemsa) before microscopic review. This workflow yields the morphological cues used for AML screening and triage and provides the reference labels that many AI systems learn from; its standardization (smear quality, staining, scanning) is critical for model generalizability.

Morphological examination of PBS and BMS is a fundamental and indispensable step in the diagnostic workflow of AML (28) (see **Figure 2**). Traditionally, this process relies on manual microscopic evaluation by hematologists, who assess various cellular features such as shape, size, color, and internal structures to determine the degree of differentiation, maturation status, and pathological abnormalities of blood cells (29). In AML, blood smears often reveal abnormal blast cells that are typically characterized by increased cell size, a high nucleocytoplasmic ratio, prominent nucleoli, reduced cytoplasmic volume, and abnormal granule distribution (30). These morphological abnormalities serve as critical indicators in the diagnosis of AML. Additionally, in certain subtypes such as APL, morphological cues may provide important subtype-specific diagnostic clues.

However, traditional morphological analysis is highly dependent on the observer’s expertise and is inherently subjective. The slide review process is labor-intensive and time-consuming (31), and even among experienced hematologists, inter-observer agreement on cell classification remains limited, with reported consistency rates of only around 60% (32).

With the advancement of automated hematological analysis, AI-driven models have been developed to automatically detect and classify leukemic cells, thereby assisting in the diagnosis of AML (33[*preprint*], 34). Recent studies have focused on the automated identification of leukemic cell morphology in PBS and BMS, subtype classification, and even the prediction of underlying genetic features.

### 4.2 Cell segmentation and feature extraction

Accurate segmentation of individual blood cells from complex smear backgrounds is a fundamental prerequisite for subsequent classification tasks. Traditional image processing methods have been widely employed for cell segmentation, including manual color thresholding (31), Otsu thresholding combined with morphological operations such as erosion and dilation for cytoplasm and nucleus segmentation (35), and K-means clustering for nucleus extraction (36). To address the challenge of overlapping cells, the watershed distance transform algorithm has proven effective for separating closely adherent leukocytes (37). In addition, more advanced techniques such as active contour models and fuzzy C-means clustering have been used to precisely delineate the boundaries of leukemic cells (38).

Compared with traditional image processing techniques, deep learning models are better equipped to handle complex backgrounds and cellular heterogeneity, thereby achieving superior performance in cell segmentation tasks. For example, Mask R-CNN has been widely applied for object detection and pixel-level segmentation of blood cells (39). introduced WBC-Net, a hybrid architecture that combines UNet++ and ResNet, significantly improving the precision of leukocyte boundary detection (40). Similarly, Roy et al. developed a semantic segmentation framework based on DeepLabv3+, which offers enhanced accuracy in delineating cell contours (41). In addition, some studies have proposed moment-based localization methods in the CMYK color space for extracting regions of interest, effectively balancing segmentation efficiency and accuracy (42).

Before inputting blood cell images into a classifier, it is necessary to extract features that can effectively distinguish between different cell types. Traditional approaches rely on manually engineered features, including geometric, color, and texture characteristics of the cells (43, 44). Geometric descriptors typically include parameters such as area, perimeter, nucleocytoplasmic ratio, and nuclear shape (45). Color features involve statistical measures such as the mean and variance of RGB or HSV color channels (46), while texture features describe the spatial distribution of structural patterns, commonly using gray level co-occurrence matrices and local binary patterns (LBP) (47).

In contrast, deep learning methods, particularly convolutional neural networks (CNN), can automatically learn hierarchical and task-specific representations directly from raw pixel data. For example, LeuFeatx, based on a fine-tuned VGG16 model, achieved a macro-average recall of 64.3% on an AML dataset, outperforming manual feature extraction methods (48). Wang et al. (49) utilized a ResNet model pretrained on ImageNet, which proved effective in extracting complex and robust features from medical images.

### 4.3 AML detection and subtype classification

Increasing research attention has been directed toward developing automated models based on image data to distinguish leukocytes from AML patients and healthy individuals, and to further perform AML detection and subtype classification (see **Table 3**). For instance, Dinčić et al. (47) utilized support vector machines (SVMs) to classify mature and immature leukocytes using manually extracted morphological, fractal, and texture features, achieving an average classification accuracy of 80%. Liu et al. (50) analyzed bone marrow smear images obtained from the TCIA database and extracted two morphological features, six radiomic features, and one clinical feature. A random forest (RF) model was then used to classify AML subtypes.

**Table 3:** Overview of research on adjunctive diagnosis of AML based on blood smear images.

Authors (year)	N(train/val/test)	Multi-institutional	Segmentation method	Feature extraction method	Classifier(s) employed	Result	External validation	Bias mitigation	Scope	Clinical endpoint assessed
Dasariraju et al. (2020) (35)	12,74 images (80/20 split)	N	Ostu thresholding, morphological operations	Morphological features	ML (RF)	Accuracy: 92.99% Sensitivity: 95.41% Specificity: 90.48% AUC: 98%	N	Y (Class balancing)	Cell classification	N
Dinčić et al. (2021) (47)	18,365 images (8-fold CV)	N	ImageJ software	Morphological features, textural features, fractal Character	ML (SVM)	Precision: 80%	N	N	Cell classification	N
Rastogi et al. (2022) (48)	18,365 images (80/20 split)	N	NR	CNN (VGG)	ML (SVM, XGBoost, RF, extra trees classifier)	Accuracy: 96.15%	N	Y (Class balancing, data augmentation)	Cell classification	N
Roy et al. (2022) (54)	18,365 images (80/20 split)	N	NR	CNN (ResNet, VGG, GoogleNet)	CNN (ResNet, VGG, GoogleNet)	F1-Score: >91% Precision: 95.74%	N	Y (Class balancing, data augmentation)	Cell classification	N
Badruzzaman et al. (2023) (109)	961 individuals (60/15/25 split)	N	NR	CNN (ResNet, EfficientNet)	CNN (ResNet, EfficientNet)	Accuracy: 78.11%, Precision: 71.94%, Recall: 75.00%, F1-Score: 73.03%	N	Y (Class balancing)	Cell classification	N
Elhassan et al. (2023) (110)	200 individuals (80/20 split)	N	CMYK- Moment	Deep convolutional autoencoder, CNN	Deep convolutional autoencoder, CNN	Accuracy: 97% Precision: 98% Sensitivity: 97% AUC: 99.7%	N	Y (Class balancing)	Cell classification	N
Park et al. (2024) (111)	42,386 images (80/20 split)	N	NR	CNN (EfficientNet)	CNN (EfficientNet)	Accuracy: 88.58% F1-Score: 73.61%	N	Y (Consensus-based Annotation, data augmentation, ensemble model)	Cell classification	N
Ouyang et al. (2021) (39)	13,504 images (9,772/2,443/1,289)	N	Mask R-CNN	Mask R-CNN	Mask R-CNN	Precision: 62.5% Recall: 84.1%	N	Y (Data augmentation)	AML classification	Diagnostic classification of APL vs non-APL
Sidhom et al. (2021) (112)	106 individuals (82/24 split)	N	NR	CNN	CNN	AUC: 89%	Y	Y (Stain normalization)	AML classification	Diagnostic classification (APL diagnosis, molecular ground-truth)
Liu et al. (2022) (50)	50 individuals (3/1 split)	N	Watershed	Morphological features, radiomics features, clinical feature	ML(RF), ANN(BLS)	Accuracy: 99.8% Precision: 100% Recall: 99.6% AUC: 99.8% F1-Score: 99.8%	N	Y (Feature selection)	AML classification	Diagnostic classification of AML subtypes (M1 vs M2)
Hehr et al. (2023) (24)	189 individuals (5-fold CV)	Y	Metafer software	CNN (ResNet)	Single-Cell based Explainable Multiple Instance Learning Algorithm	F1-Score: 86%	N	Y (Data cleaning, data augmentation)	AML classification	Diagnostic classification of AML genetic subtypes (PML::RARA, NPM1, CBFB::MYH11, RUNX1::RUNX1T1) vs healthy controls
Acharya et al. (2023) (113)	1,500 images (1000/500)	N	K-medoids, watershed, Transform	Shape features, color features, texture features	ML (RF, DT, KNN, Naive Bayes)	Accuracy: 99%	N	Y (Feature selection, stain normalization)	AML classification	Diagnostic classification of AML subtypes (M2–M5) and NRBC detection
Mustapha et al. (2025) (114)	81,214 images (70/15/15 split)	N	NR	CNN (ConvNetXT)	CNN (ConvNetXT)	Accuracy: 95%	N	Y (Data augmentation, class balancing)	AML classification	Diagnostic classification of AML genetic subtypes (CBFB::MYH11, RUNX1::RUNX1T1, PML::RARA, MLL::AF9) vs healthy
Shaheen et al. (2021) (34)	4,000 images (70/30 split)	N	NR	CNN (AlexNet)	CNN (AlexNet)	Accuracy: 98.58% Precision: 87.4% Sensitivity: 88.9%	N	N	AML detection	Diagnostic classification: AML vs non-AML
Ramya et al. (2021) (38)	18,365 images (NR)	N	Active contour−based model, Fuzzy C−mean clustering	Image level Features, statistical features.	ANN	Accuracy:96.56% Precision: 97.2% Recall: 97.9% Sensitivity:96.9% Specificity:97.81%	N	N	AML detection	Diagnostic classification of AML vs normal (image-level detection)
Wang et al. (2022) (49)	115 images (70/30 split)	Y	NR	CNN (ResNet)	CNN (ResNet)	Accuracy:92.9% AUC:96.8%	Y	Y (Data augmentation)	AML detection	Diagnostic classification of AA, MDS, and AML from bone marrow smears
Abhishek et al. (2022) (53)	500 images (80/20 split)	N	NR	LBP, HOG, CNN	SVM, CNN	Accuracy: 95%	Y	Y (Data augmentation)	AML detection	Diagnostic classification: binary (cancer vs normal) and three-class (ALL vs AML vs normal)
Venkatesh et al. (2022) (55)	22,384 images (NR)	Y	NR	CNN (ResNet)	CNN (ResNet), Meta-Learning	Accuracy: 97% Precision: 96.6% Recall: 96.55% F1-Score: 96.65%	N	Y (Few-shot learning)	AML detection	Diagnostic classification of AML vs normal vs other leukocyte classes (multi-class WBC subtype classification)
Baig et al. (2022) (115)	4,150 images (70/30 split)	Y	NR	CNN	ML (SVM, Bagging ensemble, RuSBoost, KNN)	Accuracy: 97.04%	N	Y (Data augmentation, class balancing)	AML detection	Diagnostic classification of malignant leukemia subtypes (ALL, AML, MM) from microscopic smear images
Li et al. (2023) (116)	12,466 individuals (80/20 split)	N	NR	Faster R-CNN	SVM	Accuracy: 97.16% Sensitivity: 99.09% Specificity: 92%	N	Y (Data augmentation)	AML detection	Diagnostic classification of hematologic neoplasms: normal vs abnormal, and subtype identification (e.g., AML with differentiation, APL, ALL, CML-CP, CLL, MM, MPN, aplastic anemia)
Haque et al. (2024) (52)	35,114 images (75/5/25 split)	Y	NR	CNN	ML (KNN, MLP, RF, SVM, SGD) CNN (AlexNet, ResNet, RetinaNet, CenterNet, Xception)	F1-Score: 95.89%	N	Y (Data augmentation)	AML detection	Diagnostic classification of leukemia (binary: ALL vs normal; multiclass: ALL, AML, CLL, CML, H)
Al-Bashir et al. (2024) (117)	670 images (80/15/5 split)	Y	NR	CNN (AlexNet, DenseNet, ResNet, VGG)	CNN (AlexNet, DenseNet, ResNet, VGG)	Accuracy: 94%	N	Y (Data augmentation)	AML detection	Diagnostic classification of leukemia types (ALL, AML, CLL, CML) vs normal
Boldúa et al. (2021) (51)	16,450 images (85/15 split)	Y	NR	CNN (VGG)	CNN (VGG)	Precision:93.7%, Sensitivity:100%, Specificity:92.3%	Y	Y (Data augmentation)	AML detection and classification	Diagnostic classification of acute leukemia lineage (APL, AML, ALL vs infections/controls)
Eckardt et al. (2022) (15)	1,335 individuals (NR)	Y	Faster R-CNN	CNN (Xception)	ENN	AUC: 95.85%, 85.75%	N	Y (Data augmentation)	AML detection and classification	Diagnostic classification: APL vs non-APL AML vs healthy donors,
Eckardt et al. (2022) (58)	94,162 images (4:1 split)	Y	Faster R-CNN	Computer vision algorithms	CNN (Xception, ResNet)	AUC:96.99%, 92%	N	Y (Data augmentation)	AML detection and classification	Diagnostic classification of AML vs healthy; mutation status prediction (NPM1 mut vs wt)
Kockwelp et al. (2024) (57)	408 individuals (NR)	Y	NR	CNN(ResNet)	CNN (ResNet)	AUC: 65%-93%	Y	Y (Data augmentation)	AML molecular prediction	Prediction of therapy-relevant genetics (NPM1, FLT3-ITD, CBFB::MYH11, MRC cytogenetics, ELN 2017 favorable risk)
Cheng et al. (2024) (56[*preprint*])	205 individuals (NR)	Y	Morphogo system	CNN	CNN	Accuracy: 90.63% Precision: 71.88% Sensitivity: 95.65% Specificity: 92.68%	Y	Y (Data augmentation)	AML molecular prediction	Diagnostic classification: detection of RUNX1::RUNX1T1 fusion AML from morphology

In the realm of deep learning, CNN have demonstrated strong capabilities in automatically extracting high-dimensional discriminative features from peripheral blood or bone marrow smear images. These models have achieved sensitivity and specificity exceeding 90% in AML morphological recognition tasks (33[*preprint*]). For example, Shaheen et al. (34) used AlexNet to detect AML from bone marrow images with a classification accuracy of 98%.

However, training end-to-end deep learning models often requires large annotated datasets. To address this, several studies (51–54) have applied transfer learning, where CNNs are pretrained on large-scale general-purpose image datasets such as ImageNet and then fine-tuned for specific medical imaging tasks. In addition, Venkatesh et al. (55) proposed a few-shot learning approach by integrating a pretrained ResNet with meta-learning techniques, enabling accurate AML classification from limited samples.

Model interpretability is also a critical concern, particularly in clinical applications. Hehr et al. (24) introduced SCEMILA, an interpretable AI model for AML subtype classification from blood smears. The model’s highly attentive cells showed strong agreement with diagnostically relevant cells annotated by experts. Remarkably, SCEMILA could highlight subtype-specific cells and deconstruct blood smear composition without requiring single-cell annotations, offering a valuable example of explainable AI in hematologic diagnosis.

Furthermore, several studies have explored the use of image data to predict AML-related molecular alterations. Cheng et al. (56[*preprint*]) analyzed 60,000 bone marrow smear images from 205 AML patients and successfully predicted the presence of the RUNX1::RUNX1T1 fusion gene, achieving a sensitivity of 95% and specificity of 92% on the test set. Kockwelp et al. (57) trained a ResNet model using single-cell images derived from BMS to predict mutations such as CBFB::MYH11, NPM1, and FLT3-ITD, and employed sensitivity-based heatmaps for phenotype-genotype interpretability. Eckardt et al. (58) proposed a multi-step deep learning framework that performed cell segmentation, AML classification, and NPM1 mutation prediction, achieving an AUC of 0.92.

### 4.4 Limitations and future considerations

Despite encouraging results in automated cell segmentation, feature extraction, and classification, most blood smear studies remain limited by small, single-center datasets and substantial variability in staining protocols and imaging quality. Many models rely on retrospective data and lack external validation across institutions, raising concerns about generalizability (59). While some efforts, such as interpretable frameworks (e.g., SCEMILA), demonstrate potential to enhance transparency, the majority of CNN-based models still function as “black boxes.” (24). In addition, there is little evidence of integration into clinical workflows, where turnaround time, interpretability, and cross-platform robustness are essential. These limitations highlight the gap between promising algorithmic performance and actual clinical applicability in hematopathology.

To overcome these issues, future efforts should prioritize the development of large-scale, standardized, and cross-institutional image databases, along with the design of inherently interpretable network architectures to enhance both transparency and clinical utility in AML diagnosis.

## 5 Adjunctive diagnostic of AML based on flow cytometry

### 5.1 Flow cytometry

**Figure 3:**
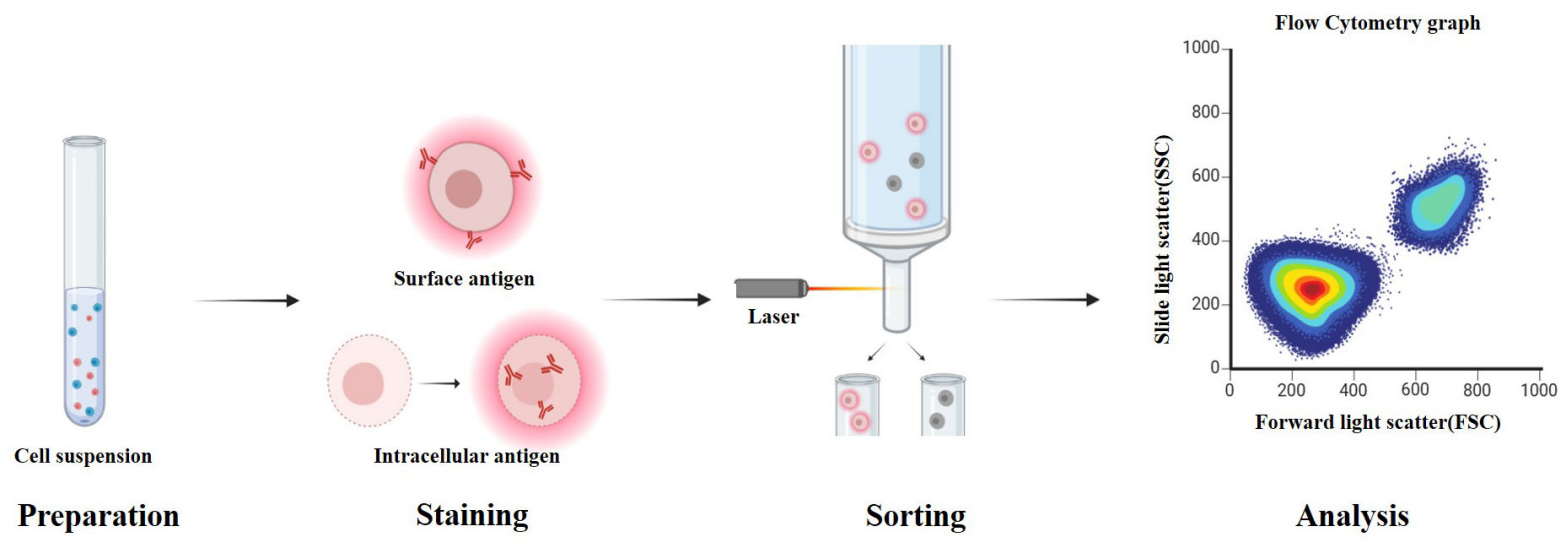
Workflow of flow cytometry for detecting cell surface and intracellular antigens. Cells are first suspended in buffer and stained with fluorescently labeled antibodies. Surface antigens bind directly to the antibodies, while intracellular antigens require fixation and permeabilization to allow antibody access to the cytoplasm or nucleus. After staining, cells pass through the flow cytometer, where lasers excite the bound fluorochromes. Forward and side scatter, along with emitted fluorescence, are measured to analyze cellular characteristics. These readouts underpin AML diagnosis and MRD assessment and are targets for AI systems that automate gating and rare-population detection; panel standardization and external validation are critical for generalizability.

Flow cytometry is a critical technique for the diagnosis and monitoring of AML (see **Figure 3**). By detecting specific surface and intracellular antigen markers, it enables the identification of the lineage, differentiation stage, and aberrant immunophenotype of leukemic cells (60). MFC, which utilizes combinations of multiple antibodies, allows for the simultaneous analysis of antigen expression profiles in tens of thousands of cells. With single-cell resolution, MFC can detect rare abnormal cell populations in bone marrow, making it particularly valuable for MRD assessment (61).

Moreover, flow cytometry also contributes to guiding targeted therapies. Antigens such as CD33 and CD123 serve not only as diagnostic markers for AML but also as therapeutic targets for antibody-based treatments (62). Compared to other techniques, flow cytometry offers rapid immunophenotyping within a few hours of sample processing and has the ability to distinguish viable cells from debris and dead cells (63).

However, the widespread clinical application of flow cytometry faces several challenges. Differences in antibody panels and data interpretation protocols across institutions hinder cross-center comparability and complicate standardization efforts. High-sensitivity detection depends on advanced cytometers and fluorescent-labeled antibodies, making individual assays relatively expensive. In addition, traditional manual gating used for data analysis is labor-intensive and subject to operator bias, especially when handling large volumes of multidimensional data (64).

### 5.2 Cell population analysis

A wide range of supervised and unsupervised machine learning algorithms have been applied to replace or assist the traditional manual gating process in flow cytometry, significantly improving the efficiency and accuracy of AML-related data analysis. Unsupervised learning techniques, in particular, have shown great value in dimensionality reduction and visualization. Nonlinear techniques such as t-SNE and UMAP project high-dimensional parameters into two-dimensional space, facilitating intuitive identification of cell subpopulations (65). Clustering algorithms like K-means and density-based methods (e.g., DBSCAN) have also been widely employed for cell classification tasks involving multiparametric data (66).

In recent years, self-organizing map (SOM) models have attracted growing attention due to their capabilities in visualization and adaptive pattern recognition. For example, one study combined SOM with XGBoost to construct a hybrid model for AML diagnosis, achieving 92.55% accuracy and 99.79% specificity on the validation dataset (67). Porwit et al. (66) further applied the FlowSOM algorithm to unsupervised clustering of erythroid precursor cells, successfully identifying 18 potentially abnormal subpopulations that provided new biological insights for diagnosis.

### 5.3 Automated diagnosis

In the field of AML diagnosis and subtype classification, several studies have demonstrated high performance. Gupta et al. (68), using 10-color flow cytometry data, integrated key markers such as CD2, CD13, and CD64 into a radar plot to distinguish typical and variant APL, as well as NPM1-mutated AML, achieving 100% accuracy in identifying typical APL. Bellos et al. (69) conducted a large-scale study involving over 36,000 patients and built an AML diagnostic model combining XGBoost and SVM, attaining 99.9% accuracy in 82% of the cases, showcasing the potential of AI in large-cohort settings.

In addition to traditional shallow machine learning approaches, recent studies have increasingly explored multi-model fusion strategies and statistical modeling techniques to improve the extraction and classification of complex flow cytometry data. For instance, Ko et al. (70) combined SVM with Gaussian mixture models, achieving a classification accuracy of 92.4% in AML patient samples. Monaghan et al. (71) further introduced a Fisher kernel-based approach to extract multiparametric features, which were then classified using SVM to distinguish APL from non-APL cases. This method also identified key features associated with overall survival, offering new insights into prognostic modeling. Additionally, Cox et al. (72) structured cellular data into graph representations, demonstrating the potential of graph-based modeling techniques for detecting abnormal cell populations.

In terms of clinical translation, efforts have been made to integrate these AI-based models into routine workflows. Zuromski et al. (67) constructed a deployable AML diagnostic platform using flow cytometry data as input, enabling automatic report generation within hospital information systems. This work provides a valuable paradigm for the clinical implementation of AI-assisted diagnostic tools.

### 5.4 MRD detection and molecular feature prediction

MRD detection is widely recognized as a key metric for assessing treatment response and predicting relapse in AML. However, conventional flow-based MRD analysis requires high sensitivity and standardization, which are often difficult to maintain in routine practice. Recently, AI models have shown potential to complement or even replace manual assessment.

The MAGIC-DR framework (73), which integrates UMAP for dimensionality reduction and XGBoost for classification, achieved strong concordance with manual MRD assessments in 25 validation samples. Moreover, it identified immature monocytic populations that were often overlooked in manual analysis, thus enhancing overall detection sensitivity. Weijler et al. (74) proposed a semi-supervised strategy based on UMAP to separate abnormal populations in MRD samples, achieving an F1 score of 79.4%, suggesting its applicability in heterogeneous clinical data.

At a higher level of application, some studies have utilized flow cytometry data to predict molecular genetic features and patient prognosis. Lewis et al. (75) developed a multi-instance learning model with an attention mechanism using only flow cytometry data as input. The model achieved an AUC of 0.96 for diagnostic classification and was capable of predicting several WHO-defined genetic abnormalities in AML, such as t (8;21), t(15;17), and NPM1 mutations. Couckuyt et al. (76) further integrated flow cytometry data with machine learning algorithms to predict two-year survival, revealing significant associations between immune subtypes, genetic features, and patient outcomes.

### 5.5 Limitations and future considerations

AI-driven approaches to flow cytometry analysis have significantly reduced the reliance on manual gating and improved MRD detection. However, current studies are largely retrospective and often conducted on heterogeneous panels and protocols, reflecting the lack of international standardization. Only a few reports demonstrate prospective or real-world clinical validation, and cross-center reproducibility remains uncertain. Moreover, most machine learning models prioritize accuracy but do not adequately address class imbalance, operator bias, or rare subpopulation detection. While pilot platforms for automated reporting exist, their clinical readiness is still low-to-moderate, requiring regulatory approval, standardized antibody panels, and better interpretability tools before widespread adoption.

Overall, AI-assisted workflows have demonstrated strong consistency with expert assessments in various tasks, including cell population analysis, AML classification, and MRD detection (see **Table 4**). Future directions should focus on building standardized, multi-center flow cytometry data platforms to improve model generalizability. In addition, developing interpretable models and enhancing visualization capabilities will be critical for clinical integration, particularly in detecting rare subpopulations and tracking dynamic changes in MRD.

**Table 4:** Overview of research on adjunctive diagnosis of AML based on flow cytometry data.

Authors (year)	N(train/val/test)	Multi-institutional	Method	Result	External validation	Bias mitigation	Scope	Clinical endpoint assessed
Patay et al. (2021) (118)	203 flow cytometry Samples(NR)	N	SOM, Neural Networks	Accuracy: 99% F1-Score: 98%	N	Y (QC via Bioconductor)	Cell Classification	Cell viability and population classification for AML therapeutic discovery platform
Vial et al. (2021) (119)	59 individuals(NR)	Y	FlowSom	Sensitivity: 69% Specificity: 85%	N	Y (Backgating, thresholds optimized)	MRD detection	MRD detection and correlation with complete remission, relapse, and induction failure
Weijler et al. (2022) (74)	146 individuals(NR)	Y	UMAP	F1-Score: 79.4%	N	Y (Marker pre-filtering, patient-level CV)	MRD detection	Clinical endpoint: MRD detection in AML bone marrow (flow cytometry, blast identification vs manual gating
Seheult et al. (2023) (120)	70 flow cytometry samples	N	PARC, UMAP	Consistency: 100%	N	Y (FlowCut error removal,	MRD detection	MRD detection in AML bone marrow by flow cytometry, correlation with manual gating and molecular MRD
Shopsowitz et al. (2024) (73)	113 samples (98/25)	Y	XGBoost, UMAP	AUC: 97%	N	Y (Ensemble methods)	Cell classification and MRD detection	AML MRD detection in bone marrow/peripheral blood; concordance with conventional flow MRD
Gupta et al. (2021) (68)	84 patients (NR)	Y	Radar plot	Accuracy: 90%	N	Y (Standardized panels, gating strategy)	AML classification	Differential diagnosis of APL vs NPM1+ AML
Cox et al. (2024) (72)	68 patients (49/19)	N	GNN	Accuracy: 100%	N	Y (Preprocessing of data, 15 random train/val splits	AML classification	Distinguishing APL vs other AML
Bellos et al. (2021) (69)	3,961 patients (80/20 split)	Y	XGBoost, SVM, AutoGluon	Precision: 99.8% Recall: 99.8%	N	Y (Feature engineering)	AML detection	Diagnostic classification of hematologic neoplasms (AML, ALL, MDS, MM, NHL subtypes)
Monaghan et al. (2022) (71)	531 patients (80/20 split)	Y	GMM, Fisher kernel method, SVM	Accuracy: 94.2% AUC: 99.5%	N	Y (Feature selection)	AML detection	Diagnostic classification: APL vs AML/not APL vs ALL vs nonneoplastic cytopenias
Zhong et al. (2022) (121)	727 samples (500/227)	N	DeepFlow software, multidimensional density–phenotype coupling algorithm, RF	Consistency: 97.1%	N	Y (QC filters)	AML detection	Acute leukemia classification (AML, B-ALL, T-ALL vs non-leukemic)
Lu et al. (2023) (122)	117 individuals (NR)	N	DeepFlow software	Accuracy: 94%	N	Y (QC through manual comparison)	AML detection	Classification of acute leukemia (AML vs ALL vs normal)
Lian et al. (2024) (123)	453 samples (70/30 split)	N	CNN, GAN	Accuracy: 86%	N	Y (Data cleaning)	AML detection	Diagnostic classification of AL (Normal vs AML vs ALL subtypes incl. BCP-ALL, T-ALL)
Müller et al. (2023) (124[*conference*])	2,400 individuals (NR)	Y	XGBoost	Precision: 99% Recall: 99%	N	Y (Expert-informed features, standardized cytometer processing)	AML detection	Diagnostic classification across hematologic neoplasms (AML, ALL, MDS, MM, B-/T-NHL)
Cheng et al. (2024) (125)	241 patients (80/20 split)	N	ResNet	Sensitivity: 94.6%	N	Y (Data augmentation)	AML detection	Diagnostic classification of AML, B-ALL, T-ALL vs normal/other,
Zuromski et al. (2025) (67)	18,379 samples (13,566/3,464/1,349)	Y	SOM, XGBoost	Precision: 92.55 Sensitivity: 76.99% Specificity: 99.79%	N	Y (Feature selection)	AML detection	AML detection in triage flow cytometry panels
Couckuyt et al. (2025) (76)	122 patients (NR)	N	FlowSoM, XGBoost	Accuracy: 72%-88%	N	Y (Feature selection)	AML prognosis prediction	Relapse, 2-year survival, ELN risk, NPM1 mutation, inv(16) prediction
Lewis et al. (2024) (75)	1,820 samples (80/20 split)	Y	Attention-Based Multi-Instance Learning Models	Accuracy: 92.2% AUC: 96.5%	N	Y (5-fold CV, class balancing)	AML detection and molecular prediction	Diagnostic classification (Acute leukemia vs non-leukemia; AML vs ALL); prediction of cytogenetic/molecular variants (e.g., PML::RARA, RUNX1::RUNX1T1, NPM1)

## 6 Adjunctive diagnostic of AML based on genetic data

### 6.1 Genetic analysis

Genetic analysis provides essential molecular insights that play a pivotal role in disease classification, risk stratification, and therapeutic decision-making in AML (77). Traditional approaches such as karyotyping and fluorescence *in situ* hybridization (FISH) have long been utilized in clinical diagnostics. In recent years, the emergence of advanced genomic technologies, including next-generation sequencing (NGS), single-cell sequencing, and transcriptome analysis, has continuously propelled the advancement of precision medicine in AML (78).

Karyotyping primarily uses G-banding to detect numerical and structural chromosomal abnormalities, such as trisomies, deletions, inversions, and translocations (79). FISH, on the other hand, utilizes fluorescent probes to identify specific gene rearrangements or chromosomal region abnormalities with higher resolution. It is commonly used to detect fusion genes such as PML::RARA and RUNX1::RUNX1T1 (26).

**Figure 4:**
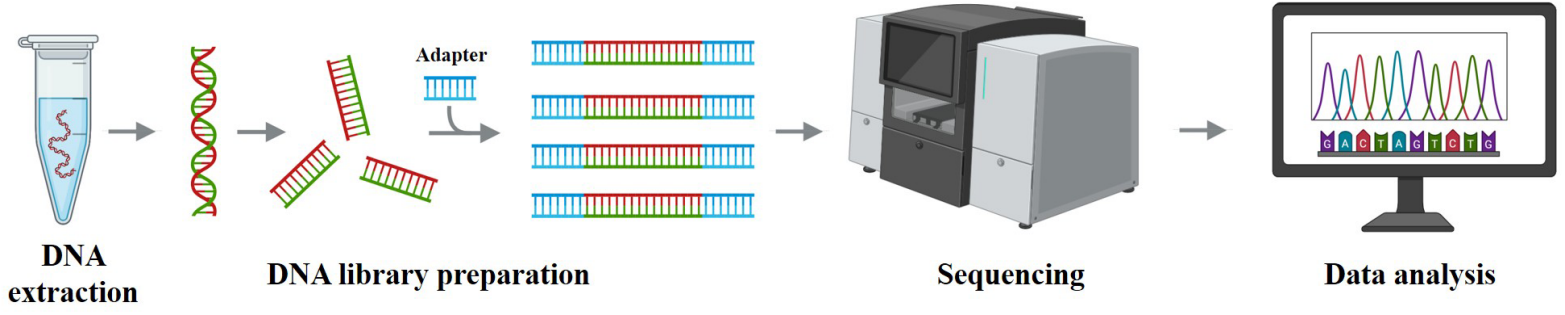
Schematic workflow of next-generation sequencing. Genomic DNA is extracted, fragmented, and ligated to adapters to build libraries, which are sequenced on a high-throughput platform; reads are then aligned and variants are called and annotated. These steps generate the molecular features used for AML diagnosis/risk stratification and for training AI models; pipeline harmonization (variant-calling/batch-effect control) and external multi-cohort validation are essential for generalizable results.

At the molecular level, NGS is a high-throughput sequencing technology (80) (see **Figure 4**) that enables comprehensive analysis of genomic or exonic mutations, insertions, and deletions, and has been widely applied in AML subtyping and prognostic evaluation. Single-cell sequencing allows the profiling of gene expression or genomic variations at the single-cell level, enabling the identification of leukemic subpopulations at different differentiation stages and offering insights into key subclones and relapse mechanisms (77). Transcriptome sequencing provides a global gene expression profile of AML patients, helping to identify expression signatures, dysregulated pathways, and prognostic biomarkers associated with AML development (81).

Despite advances in genetic testing methodologies, challenges remain. Traditional cytogenetic methods such as karyotyping and FISH are limited in throughput, sensitivity, and turnaround time (82), while modern techniques like NGS and single-cell sequencing are constrained by high costs, complex data interpretation, and a heavy reliance on bioinformatics expertise (26). With ongoing technological development, the integration of diverse genetic data combined with AI and machine learning holds promise for enhancing diagnostic accuracy and enabling more personalized treatment strategies for AML.

### 6.2 Karyotyping and FISH

Karyotyping is a standard technique for detecting chromosomal abnormalities. Traditionally, the interpretation of chromosome images requires highly experienced cytogeneticists. To enhance efficiency and reduce manual workload, recent studies have explored the use of AI to automate metaphase image recognition and chromosome classification (83), as well as to develop models for automated chromosome segmentation and pairing (84). For example, Hu et al. (85) proposed a multilayer CNN combined with a Softmax classifier, achieving 93.8% accuracy in pairing and identifying abnormal chromosomes. Similarly, Vajen et al. (86) developed a CNN-based tool that achieved 98.8% accuracy in chromosome classification and reduced manual analysis time by up to 42%.

Most current AI systems for karyotyping depend on large annotated datasets. To address this limitation, one study (87) proposed a machine learning strategy to simulate abnormal karyotype images from normal ones, combining this approach with a ResNet classifier that achieved over 95% accuracy. Furthermore, Shamsi et al. (88[*preprint*]) introduced the Vision Transformer (ViT) architecture into karyotype analysis for the first time, developing an end-to-end model that accurately identified clinically significant abnormalities such as t(9;22) from metaphase images. This approach significantly reduced the need for extensive labeled data by employing pre-training and fine-tuning strategies.

FISH is another widely used technique for detecting chromosomal number and structural abnormalities using DNA-targeted probes. However, traditional FISH analysis is labor-intensive and highly reliant on expert interpretation. To improve throughput and consistency, researchers have applied AI-based models to automate the FISH image analysis pipeline. Gudla et al. (89[*conference*]) developed a CNN-based system for automated detection of chromosomal abnormalities, achieving an accuracy rate exceeding 98%. Xue et al. (90) constructed an end-to-end detection model combining YOLOv3 with ResNet18 to assess gene amplification status, reaching 85% classification accuracy on the test set. Xu et al. (91) further proposed a multi-scale MobileNet-YOLOv4 framework for rapid detection of genetic abnormalities in circulating cells, achieving 93% accuracy and up to 500-fold improvement in detection speed. In addition, Bouilhol et al. (92) introduced DeepSpot, a deep learning tool designed to enhance the detection of fluorescent signals in single-molecule FISH images, attaining an accuracy of up to 97%.

### 6.3 Molecular analysis and prognostic prediction

Molecular genetics testing represents a core component of AML diagnostics, providing critical insights that inform classification, prognosis, and therapeutic decision-making. Mutations in genes such as NPM1 and FLT3-ITD, as well as fusion events like PML::RARA, are now incorporated into major clinical guidelines as essential molecular indicators for diagnosis and risk stratification (93).

With the rapid advancement of NGS, these techniques have increasingly been applied in clinical settings and have become indispensable tools for molecular subtyping of AML. NGS platforms can integrate whole-genome sequencing, exome sequencing, and RNA sequencing to simultaneously analyze hundreds of leukemia-related genes in a single assay, greatly improving detection efficiency and data richness (94). Wurm et al. (95) reported that the turnaround time for NGS-based analysis of AML samples decreased from 22 days in 2013 to just 10 days in 2023, reflecting substantial improvements in clinical feasibility. Zhang et al. (96) combined targeted RNA sequencing with a naïve Bayes classifier to perform differential diagnosis across 20 hematologic malignancies and 24 solid tumors, achieving an AUC of 88% for AML classification.

In large-scale applications, the integration of transcriptomic data with machine learning models has become an emerging trend in the adjunctive diagnosis of AML. Warnat-Herresthal et al. (97) integrated transcriptomic profiles from 12,029 samples across 105 studies and developed a machine learning model capable of distinguishing AML, MDS, and other myeloid neoplasms, achieving over 92% subtype classification accuracy across multiple datasets. Similarly, Angelakis et al. (98 [*preprint*]) used a CatBoost classifier on 12,708 transcriptomes from 5,052 individuals, reaching an AUC above 99% in distinguishing AML from healthy controls highlighting the synergy between big data and machine learning.

In the area of molecular subtyping and prognostic modeling, Awada et al. (99) applied Bayesian unsupervised learning to integrate mutation and immunophenotypic data, identifying four novel subtypes with distinct biological and prognostic characteristics. Song et al. (100) proposed an unsupervised multi-omics integration approach that stratified AML into three major subgroups using TCGA and clinical cohorts, showing strong generalizability. Afroz et al. (101) introduced the omicsGAN framework, which enhances predictive accuracy by synthesizing gene activity and DNA methylation profiles.

Given the high clonal heterogeneity of AML, single-cell RNA sequencing (scRNA-seq) has emerged as a powerful method to resolve cellular subpopulations and microenvironmental interactions (102). Galen et al. (103) combined scRNA-seq and genotyping data from 38,410 cells across 40 AML bone marrow samples and used machine learning to successfully classify distinct malignant subtypes and link them to specific genetic mutations. Nicora et al. (104) and Asimomitis et al. (105) applied supervised deep learning approaches to scRNA-seq data for cell state prediction and mutation status classification, respectively. Their models achieved classification AUCs of up to 98% and 84%, highlighting the significant potential of integrating single-cell omics with AI for clinically relevant analysis.

Recent studies (106) have further emphasized the role of transcriptomic changes in identifying novel therapeutic targets in AML, helping bridge the gap between genomic insights and clinical application. For instance, Gimeno et al. (107) employed a multidimensional module-optimized machine learning algorithm using RNA-seq data to predict gene mutations and drug response, providing valuable support for precision medicine. Qin et al. (108) integrated bulk RNA-seq, single-cell expression profiles, and matched clinicopathological data to construct a six-gene programmed cell death index capable of predicting chemotherapy resistance, drug sensitivity, and poor prognosis in AML patients.

With advances in high-throughput omics technologies and the integration of AI, molecular diagnostics for AML are evolving from single-marker identification toward a comprehensive, data-driven precision framework (see **Table 5**). NGS enables broad and efficient detection by combining genomic, exomic, and transcriptomic information. Transcriptomic data, when coupled with machine learning, have demonstrated exceptional performance in disease classification, subtype distinction, and prognostic assessment. Meanwhile, scRNA-seq offers unprecedented resolution of AML clonal heterogeneity and immune microenvironment features, enriching our understanding of disease mechanisms. More recently, the integration of omics data with drug response modeling has laid a foundation for individualized therapy and resistance prediction.

**Table 5:** Overview of research on adjunctive diagnosis of AML based on flow genetic data.

Authors (Year)	N(train/val/test)	Multi-institutional	Method	Result	External validation	Bias Mitigation	Scope	Clinical endpoint assessed
Shamsi et al. (2025) (88[preprint])	45,815 karyograms (42,049/3736)	Y	ViT	AUC: 94%	Y	Y (Pretraining, entropy filtering, data augmentation	Chromosome classification	Detection of chromosomal abnormalities (del(5q), t(9;22), inv(16), inv(3), t(9;11), t(11;19), PML::RARA) relevant for AML, ALL, CML, MDS diagnosis and prognosis
Fang et al. (2024) (126)	10,000 specimens (NR)	Y	Transformer	Accuracy: 100%	Y	Y (Pretraining, data augmentation)	Chromosome classification	Detection of chromosomal aberrations (del(5q), inv(3), inv(16), t(9;22), t(9;11), t(11;19)) relevant for AML, CML, MDS diagnosis/prognosis
Nicora et al. (2021) (104)	1,051 cells (705/946)	N	SVM, LR	Accuracy: 80%	Y	Y (Scanpy preprocessing)	Cell classification	Single-cell classification of malignant vs benign cells in AML bone marrow (scRNA-seq)
Asimomitis et al. (2023) (105)	50,026 cells (NR)	Y	Feedforward Neural Network	Accuracy: 98% Precision: 98% Recall: 99% AUC:>96%	N	Y (Scanpy preprocessing,normalization)	Cell classification and AML Molecular Prediction	Binary: malignant vs WT single cells; Multi-label: prediction of hotspot mutations (IDH1/2, NRAS, KRAS, NPM1, SRSF2, DNMT3A) and chromosomal abnormalities
Shah et al. (2023) (127)	1,707 patients (70/30 split)	Y	RF, XGBoost, SVM	Accuracy: 99.58% Precision: 95.77% Sensitivity: 95.77% Specificity: 99.78% F1-Score: 96%	N	Y (Feature selection, stratified CV)	AML classification	Diagnostic classification of pediatric AML molecular subtypes (e.g., KMT2Ar, NPM1, RUNX1::RUNX1T1, CBFB::MYH11, etc.), aiding risk stratification
Orgueira et al. (2021) (128)	699 patients (562/137)	Y	RF	C-index: 69.88%	Y	Y (Rank-normalization of gene expression, variable importance pruning)	AML prognosis prediction	overall survival prediction, stratification of high-risk AML patients (e.g., TP53, RUNX1, ASXL1)
Qin et al. (2024) (108)	527 patients (129/398)	Y	ML	C-index: 68%-72% AUC: 77%-81%	Y	Y (Rank-normalization, feature selection)	AML prognosis prediction	overall survival prediction in AML, prognostic stratification for therapy guidance
Afroz et al. (2024) (101)	173 samples (NR)	N	GAN	AUC: 68.78%-73.22%	N	Y (Data augmentation)	AML prognosis prediction	Predicted AML cancer phenotype/outcomes, identified significant genes, and screened candidate drugs
Song et al. (2025) (100)	481 patients (90/1,391)	Y	unsupervised multi-omics classification system	C-statistic: 87%	Y	Y (Batch effect correction)	AML classification and prognosis prediction	Overall Survival, Event-Free Survival, Complete Remission, Drug sensitivity
Cheng et al. (2022) (81)	655 patients (NR)	Y	Enhanced consensus clustering, AutoML	Accuracy: 95%	Y	Y (Batch effect correction)	AML classification and AML prognosis	Overall Survival (OS) prediction and risk stratification in AML
Wang et al. (2024) (129)	61 samples (NR)	N	Clustering algorithms	Kappa: 67.7%-68.2%	N	Y (Rigorous gating, blinded operator reading)	AML classification and AML prognosis prediction	MRD positivity as surrogate endpoint for relapse-free survival (RFS) and overall survival (OS)
Lee et al. (2021) (130)	439 individuals (12/427)	N	SVM	Accuracy: 97.2% Sensitivity: 99.5% Specificity: 98.7%	Y	Y (Leave-one-out cross-validation)	AML detection	Diagnostic classification: AML vs B-ALL vs MPAL; detection of BCR-ABL1 and novel MAP2K2 fusion; identification of mutations
Zhang et al. (2023) (96)	5,450 patients (3,045/1,415)	Y	Geometric mean naïve Bayesian	Accuracy: 88%	N	Y (Leave-one-out cross-validation)	AML detection	Differential diagnostic classification across 47 hematologic & solid tumor entities
Angelakis et al. (2024) (98[*preprint*])	5,052 individuals (80/20 split)	Y	CatBoost	AUC: 99%	N	Y (Feature selection, class balancing)	AML detection	Diagnostic classification: AML vs Healthy and AML vs Healthy+Other diseases
Yeung et al. (2024) (131)	48 patients (NR)	N	CytoTerra cloud-based analysis platform	Concordance: 100%	N	Y (QC of libraries, Blinded analysis)	AML risk stratification	Cytogenetic risk variants per ELN 2022

### 6.4 Limitations and future considerations

Genetic and transcriptomic studies represent one of the fastest-growing areas, especially with the advent of NGS and single-cell sequencing. Nonetheless, many published works face challenges of small sample size, high-dimensional data with risk of overfitting, and a heavy reliance on bioinformatics expertise. Several cited studies are still preprints, reflecting the novelty but also the limited peer-reviewed validation of these methods. External, multi-cohort validation is rare, and clinical integration of AI-based omics prediction frameworks is currently exploratory rather than routine. Furthermore, the complexity of multi-omics integration and the lack of interpretability hinder clinical decision-making.

Thus, omics-based AI models hold promise for risk stratification and prognostication. Looking ahead, intelligent clinical decision platforms that integrate omics, algorithmic inference, and clinical workflows are poised to facilitate the implementation of precision medicine in AML (78).

## 7 Conclusions and clinical integration

The pronounced heterogeneity of AML poses substantial challenges for both diagnosis and prognostic assessment. In recent years, AI has demonstrated remarkable potential to address these challenges by integrating flow cytometry data, medical images, and multi-omics information. Across diverse studies, AI models have achieved strong performance in key tasks such as cell-population identification, subtype classification, molecular-mutation prediction, and prognostic stratification. Research has also evolved from single-modal classification toward multimodal data fusion and molecular feature modeling, with increasing emphasis on interpretability and automated integration.

Despite these advances, AI still faces major barriers to clinical translation, including data heterogeneity, limited model generalizability, high annotation costs, and the scarcity of prospective validation. Future work should prioritize building standardized data platforms, enhancing model robustness and interpretability under real-world variability, and enabling seamless integration of AI systems into diagnostic and therapeutic processes. With continued innovation, AI is expected to become a core component of precision AML care, providing efficient and individualized decision support.

Clinical integration and workflow impact are essential for realizing this potential. In practice, deep learning models must be embedded into existing diagnostic and treatment pathways. During triage and diagnosis, they can rapidly screen peripheral blood smears and prioritize suspected AML cases, accelerating identification of high-risk patients. For urgent subtypes such as APL, models can trigger seconds-level alerts to facilitate timely intervention. Automated analysis substantially shortens morphological review time, a critical metric in emergency leukemia management. In measurable residual disease monitoring, sensitivity thresholds allow detection of very low abnormal-cell fractions, while cross-validation with flow cytometry or molecular assays reduces false positives and enhances longitudinal reliability. Model outputs—including calibrated confidence scores and visual heatmaps—should be standardized for interoperability with hospital information systems, supporting efficient hematopathologist review. When discrepancies arise between automated and manual interpretations, conflict-resolution strategies such as double-blind review, expert-panel adjudication, or weighted consensus voting ensure quality control. Within this closed-loop framework, AI can evolve into a “trustworthy, traceable, and controllable” collaborator that augments clinical expertise without replacing it.
